# Contribution of the 5G Smart First-Aid Care Platform to Achieving High-Quality Prehospital Care

**DOI:** 10.2196/43374

**Published:** 2023-05-22

**Authors:** Tao Xiang, Pei Yong Zhang, Guang Ying Zhuo, Hang Dai

**Affiliations:** 1 Department of Emergency The Third People’s Hospital of Chengdu Chengdu, Sichuan China

**Keywords:** fifth generation, 5G, prehospital first-aid care, smart medical care, telemedicine

## Abstract

China is gradually becoming an aging society, and the necessity for prehospital first-aid care is increasing. However, there is a long-term information blind spot in traditional prehospital first-aid care. Fifth-generation (5G) network has the advantages of enhanced broadband, multiple connections, and low latency. Combined with the current prehospital first-aid system, the 5G smart medical prehospital first-aid care model creates a new opportunity for the development of prehospital first-aid care. This paper aimed to describe the 5G smart first-aid care platform and offers practical insights into the construction and application of the 5G smart first-aid care platform in small- and medium-sized cities. We first introduced the working principle of the 5G smart first-aid care platform and then chose patients with prehospital chest pain as an example to describe the whole workflow in detail. The application of the 5G smart emergency-care platform is at the stage of pilot exploration in large- and medium-sized cities. Big data statistical analysis of the completed first-aid care tasks has not been performed yet. The 5G smart first-aid care platform realizes real-time interconnection of information between the ambulance and the hospital, performs remote consultation, shortens the treatment time, and enhances treatment efficiency. Future research should focus on quality control analysis of the 5G smart first-aid care platform.

## Introduction

In China, a country with a large population, population aging is becoming increasingly obvious. It is expected that the average life expectancy will be extended to 79 years in 2030 [1], and the necessity for prehospital first-aid care will also increase. Prehospital care is the first-line treatment to complete the task of saving lives and helping patients. It is the first stop for emergency and critically ill patients, gaining time and creating treatment conditions for in-hospital treatment [2].

At present, various prehospital first-aid care modes and standards have been established in various regions according to their actual conditions. Worldwide, there are 2 models of prehospital first-aid care: the Anglo-American model and the Franco-German model. In the Anglo-American model, emergency medical technicians and emergency physicians perform on-site emergency-care tasks; after simple treatment, patients are quickly sent to the hospital [3]. The Franco-German model of rescue measures is mainly performed on-site, dispatching doctors and equipment to the scene, hoping that patients will receive high-level medical care before arriving at the hospital [4]. The prehospital first-aid care model in China is generally similar to the Anglo-American model, but it is generally composed of medical personnel [5]. However, at present, there are also a variety of sources of emergency medical personnel, including professional prehospital emergency-care teams and other departments that rotate the medical staff, but the level of professional skills is unbalanced [6]. In addition, there is a long-term information blind spot in traditional prehospital first-aid care. After receiving the visiting mission, medical institutions should not only dispatch first-aid vehicles for the first time but also hope to immediately acquire more information about the patients’ vital signs, prehospital conditions, and important examinations. Then, with the rapid development of network technology and the emergence and application of fifth-generation (5G) technology since 2019, the 5G smart medical prehospital first-aid model has been gradually put into practice in many big cities in China [7]. It has created a new opportunity for the development of prehospital first-aid care.

5G network technology has many advantages, such as increased capacity, reliability, coverage, connection density, and energy efficiency while reducing latency [8,9]. Through the digital transformation of the 5G-enabled ambulance and equipment information integration, the prehospital 5G smart first-aid care platform has been successfully constructed. Real-time video and important inspection data are sent back to the emergency command center of the emergency department. Combined with virtual reality (VR) technology equipment, hospital specialists can observe the on-site situation in the command center, exchange information with the ambulance medical staff in real time and guide the prehospital emergency treatment. At the same time, through the functions of one-click early warning, emergency reception reminder, and opening the green channel in the hospital, the hospital doctors can (1) prepare in advance or inform the specialists in the hospital to consult the emergency department in advance when the patients are being transported to the hospital and finally move the treatment forward; (2) realize that the patients are “admitted to the hospital as soon as they get on the bus”; (3) shorten the specialist treatment time; enhance the prehospital treatment quality of acute and critical diseases; and (4) improve the rescue success rate of patients [10]. The primary aim of this article is to demonstrate the importance of the 5G smart first-aid care platform and offer practical insights into the construction and application of the 5G smart first-aid care platform in small- and medium-sized cities.

## Origin of the 5G Smart First-Aid Care Platform

Relying on the accumulation of technical and business knowledge in the field of medical IT for many years, Suzhou Madison Medical Science and Technology Co, Ltd, successfully developed a complete integrated solution for emergencies and emergency medical specialty in 2022 and pioneered its use in major hospitals in China, focusing on the construction of the information system and treatment network of first aid and the emergency and specialist center; fulfilling the purpose of patient-centered care; providing information and realizing the optimization of the green channel for the treatment of critically ill patients; and providing valuable time for rescuing patients.

## 5G Network Integration of the Car-Connected Information System

Based on the digital upgrade of the ambulance, the 5G network information system of vehicles is established through the modification and integration of the vehicle integrated terminal, vehicle communication unit, vehicle display terminal, wireless headset, and vehicle video camera equipment, which supports the information collection and data integration of vehicle medical equipment such as monitors, electrocardiograms, ventilators, and point-of-care testing and is based on wireless communication and GPS technology. Finally, it realizes the remote transmission and sharing of patients’ vital signs, emergency-care medical record data, patient medical data, and video data with the hospital emergency-care command center.

## 5G Smart Medical Emergency Care

Based on the ultra-high speed and low-delay transmission of 5G mobile communication, hospital specialists have a real-time view of 4K ultra-high-definition images in the ambulance and immersive ambulance scene through 360° VR panoramic camera and VR glasses and through real-time treatment guidance and timely feedback viewing, can realize remote consultation, and can provide the whole process support for the treatment of patients, subsequently enhancing medical treatment quality and rescue success rate of major emergency diseases. At the same time, through the functions of one-click early warning, the rapid establishment of the green channel in the hospital, and emergency reception reminder, process optimization and information accessibility of the green channel can be implemented for the treatment of acute and critically ill patients, securing valuable time for rescuing patients.

## Fully Structured Prehospital Emergency Electronic Medical Record

**Figure 1:**
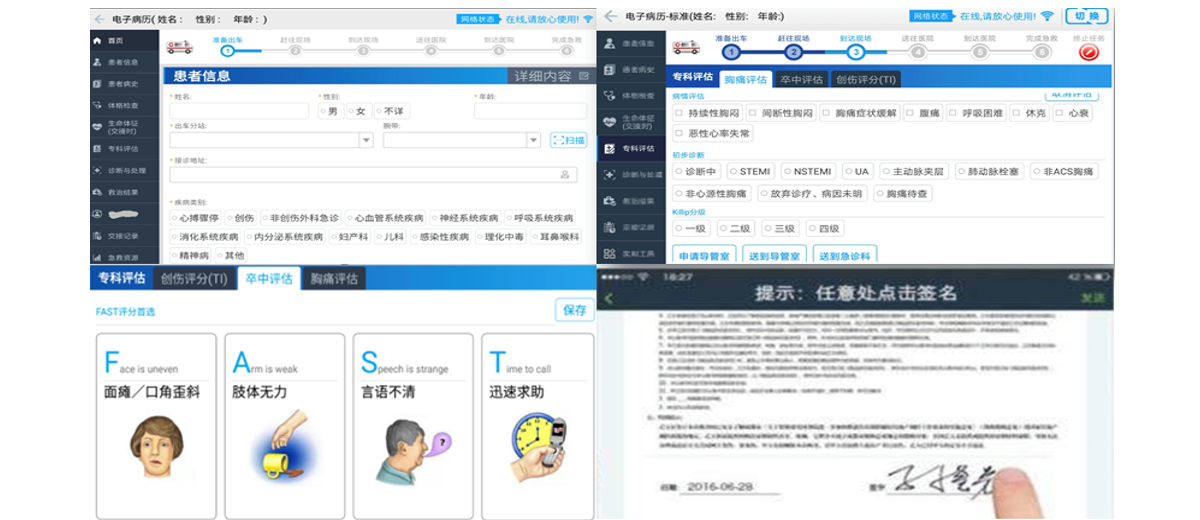
Fully structured prehospital first-aid electronic medical record. A mobile platform serves as the carrier of the electronic medical record. (A) Standard first-aid care electronic medical records, which mainly include patient information, patient history, physical examination, vital signs (handover), diagnosis and treatment, treatment results, and handover records. (B and C) Specialist evaluation: The system supports the provision of specialist evaluation tools for patients with chest pain, trauma, and stroke to achieve a specialist evaluation of these 3 types of patients. Relevant evaluation information, such as stroke score, chest pain assessment, and trauma score, is transmitted to hospital specialists in real time after the evaluation is completed to assist specialists in making rapid and accurate diagnoses. (D) Electronic handover: The system supports the electronic signature function of the “patient informed consent form” and “prehospital handover form” to realize electronic handover.

Through the rapid switching between standard emergency-care electronic medical records and multitemplate specialist electronic medical records, prehospital medical staff can flexibly collect and record patients’ condition, physical examination, diagnosis and treatment, treatment results, and other information, and the electronic signature for patients’ informed consent and prehospital handover form can also be obtained. Finally, this process quickly realizes electronic handover of prehospital emergency care (Figure 1).

## Whole Process Quality Control Management

**Figure 2:**
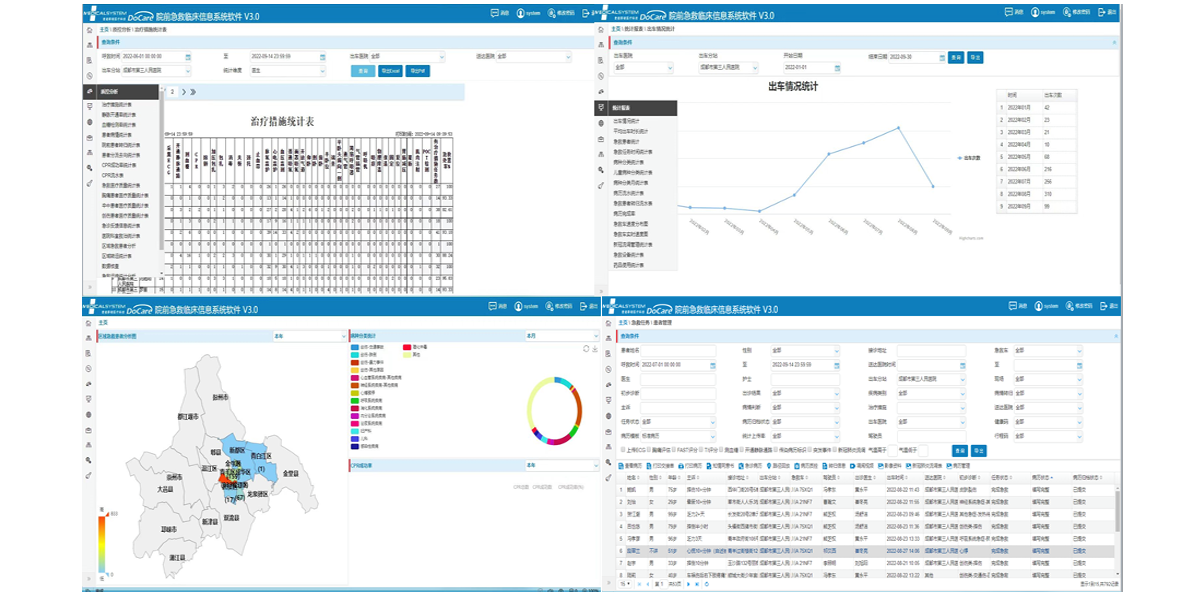
Quality control management of prehospital first-aid care. (A) Statistical table of treatment measures in quality control analysis. (B) Statistics on the number of cars in the statistical report. (C) Regional first-aid patient analysis chart, such as disease classification statistics and CPR success rate. (D) Patient management in first-aid tasks. CPR: cardiopulmonary resuscitation.

Fully structured electronic medical records combined with big data statistical analysis allow data statistical analysis and early warning functions in all dimensions of an emergency-care business, such as disease classification statistics, outcome analysis, cardiopulmonary resuscitation success rate, and emergency-care medical quality analysis, realizing the whole process quality control management of prehospital first-aid care (Figure 2).

## The Whole Workflow of the 5G Smart First-Aid Care Platform

**Figure 3:**
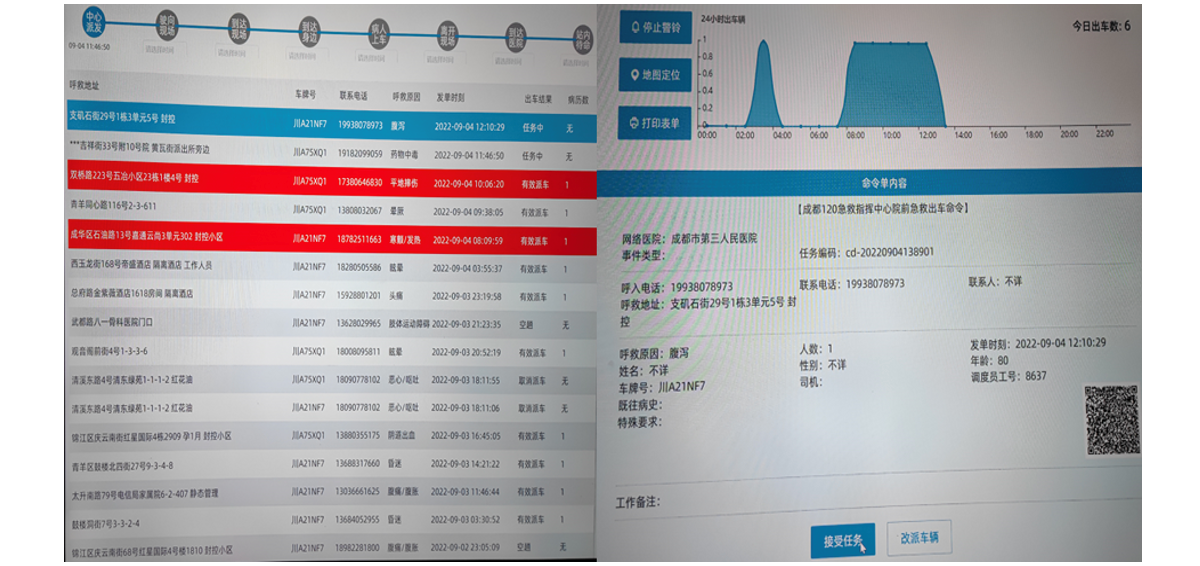
Seamless connection between the 5G smart first-aid care platform and 120 emergency dispatch system. A Chengdu 120 emergency command center prehospital first-aid dispatch order is shown. 5G: fifth generation.

After the patient calls the 120 alarm, the 5G smart first-aid care platform can directly extract the basic information of the patient from the 120 scheduling system, including the patient’s gender, age, call reason, and call location, and can realize the automatic dispatch of the scheduling task (Figure 3). The prehospital emergency-care command center is located in the emergency departments of various network hospitals, and specialists in the hospital are on standby 24/7 to provide medical guidance.

**Figure 4:**
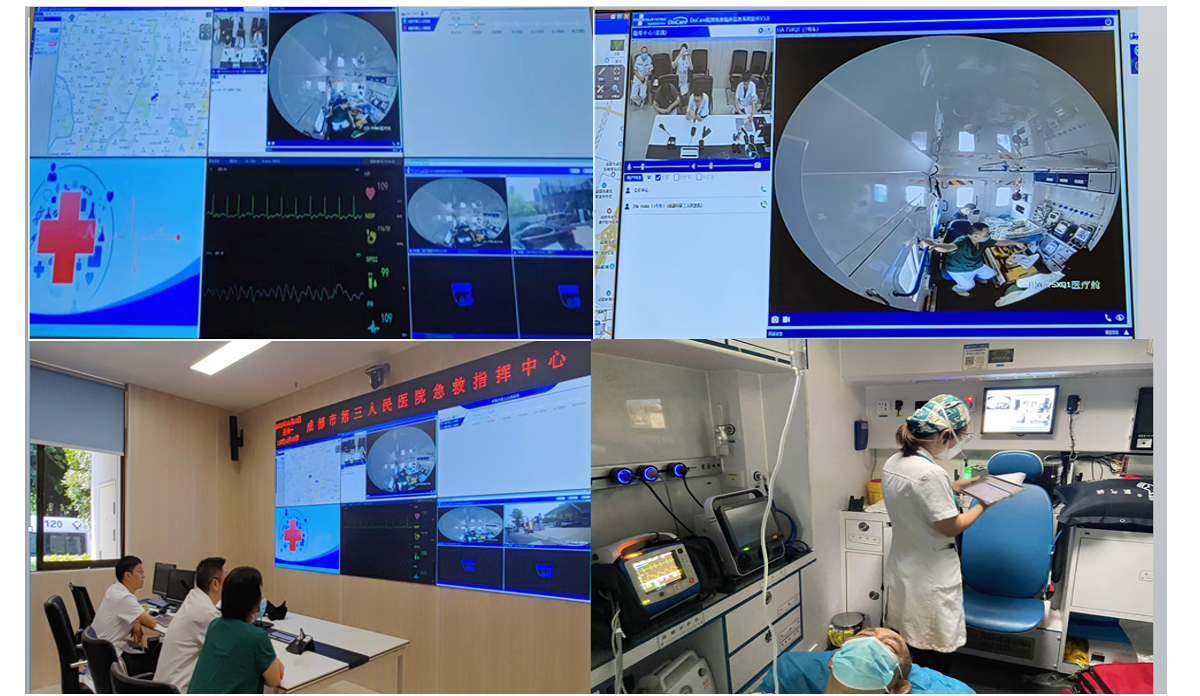
5G working diagram of the smart first-aid care platform. 5G: fifth generation.

When receiving the prehospital first-aid task, the visiting medical staff should immediately turn on the vehicle integrated terminal, vehicle display terminal, vehicle communication unit, and vehicle video camera equipment and monitor after boarding the ambulance; check the vehicle medical equipment such as electrocardiogram machine and point-of-care testing; connect the wireless headset; and be ready for a video call with the hospital specialists. When the visiting doctor transfers the patient to the ambulance from the call location, the onboard monitoring equipment is immediately connected to the patient, and the patient’s vital signs and electrocardiogram are automatically transmitted to the large screen of the hospital emergency command center. The visiting doctor reports the patient’s condition, relevant physical examination, and current treatment and disposal effect to the command center expert through the wireless headset, and the command center expert uses a 360° VR panoramic camera and VR glasses to observe the situation in the ambulance in real time, monitors the patient’s vital signs and illness changes, and provides real-time guidance on the current treatment and possible critical conditions through the wireless intercom system (Figure 4).

## An Example Case of a Patient With Prehospital Chest Pain

**Figure 5:**
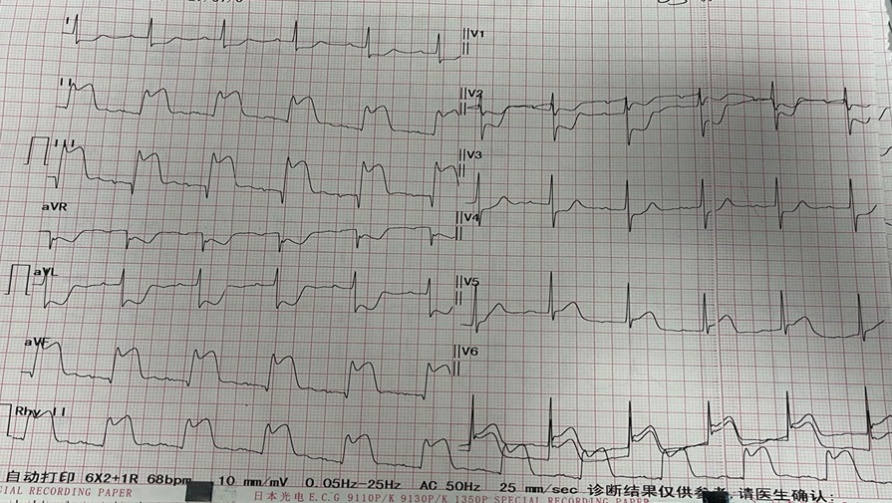
Electrocardiogram of a patient with acute myocardial infarction.

We chose a patient with prehospital chest pain as an example to illustrate the advantages of the 5G smart emergency-care platform. At 10:33 AM, Chengdu Third People’s Hospital received an assignment from the dispatching center. The patient was a 68-year-old man experiencing chest pain. At 10:40 AM, the personnel transferred the patient from his home to a 5G-enabled ambulance. The vital signs and electrocardiogram (Figure 5) of the patient were collected by the emergency personnel at 10:41 AM and uploaded to the hospital emergency command center. Through the onboard GPS and audio and video communication system, specialist consultation in the hospital was realized, and prehospital evaluation was completed ahead of schedule. The following in-hospital specialist instructions were provided. The patient’s electrocardiogram indicated inferior myocardial infarction, and venous access was established for rehydration to maintain the stability of blood pressure. A medicine packet (enteric-coated aspirin tablets, 300 mg; ticagrelor tablets, 180 mg; and atorvastatin calcium tablets, 20 mg) for myocardial infarction was given immediately. Moreover, the medical staff in the ambulance was reminded to be alert to the possibility of malignant arrhythmia or even sudden death of the patient at any time. At 10:46 AM, one-click early warning and emergency reception reminder were activated in the ambulance, the green channel in the hospital was initiated, and the catheterization laboratory in the hospital was informed to prepare for treatment. At 10:48 AM, the patient arrived at the hospital and bypassed the emergency department. At 10:50 AM, the patient successfully underwent percutaneous coronary intervention in the catheterization laboratory. At 11:50 AM, the doctor finalized the patient’s medical record.

## Discussion

Prehospital first aid may be required at any time, especially for critically ill patients, so it is crucial to make clinical decisions quickly and accurately. Prehospital first-aid care plays a vital role in the treatment of clinical emergencies such as cardiac arrest [11], stroke [12], acute myocardial infarction [13], and respiratory failure [14]. Early diagnosis and early treatment are likely to achieve better prognoses and reduce mortality and morbidity [15]. Ambulances are an important setting to start life-saving treatment before the hospital and can remotely assist in rational decision-making for the prehospital first-aid team before patients enter the hospital. Rehman et al [16] and Mukhopadhyay et al [17] applied wireless communication technology to the ambulance service. Nevertheless, the use of traditional wireless communication technologies in ambulances needs to overcome several technological challenges, such as limited, unreliable, and low-quality communication capability. Compared with previous generations of network technology, 5G has increased capacity, reliability, coverage, connection density, and energy efficiency while reducing latency. As the 5G network has emerged, the realization of a 5G-enabled service will have the potential to bring great benefits to medical emergency services. The 5G smart first-aid care platform can realize real-time supervision of the frontline doctors in the prehospital ambulance by the specialists in the hospital and real-time guidance so as to facilitate the treatment of some critically ill patients in advance and avoid possible irreversible consequences. Finally, high-quality prehospital rescue can be achieved, and the success rate of rescue is improved.

The aforementioned chest pain cases are only representative of tens of thousands of cases. With the patient-centered chest pain green channel, the 5G smart first-aid care platform advances the entire first-aid care process and completes the collection and transmission of vital signs and electrocardiogram on the vehicle side. With the help of the 5G network and audio and video technology, consultations with doctors in the hospital are completed, the quality of prehospital treatment is enhanced, and the time for specialist treatment is shortened. At the same time, when patients enter the chest pain green channel, they are fitted with a wristband that automatically records the key time points. The holographic data of the patient’s treatment, diagnosis, and treatment can be extracted, including prehospital first-aid care, and the information of each time point in the hospital forms the chest pain data for analysis in the center to provide a basis for continuous improvement of the chest pain.

The prehospital first-aid care uses the 5G smart first-aid care platform, which fully embodies the prehospital first-aid care mode of “admitted to the hospital as soon as they get on the bus,” realizes the seamless link between prehospital and the hospital, greatly shortens the rescue response time, and strives for improved health of patients. In Japan, only the ordinary prehospital first-aid care mode enables patients to receive professional medical assistance 4-7 minutes in advance [18]. A system review [19] also supports the efficiency of prehospital first-aid care using the telemedicine system in ambulances. Many studies [20-22] have also discovered that the use of telemedicine can complete prehospital diagnosis ahead of time, reduce treatment time, have higher diagnostic accuracy, and improve the level of treatment before admission [23,24]. Kim et al [25] also demonstrated, through a systematic review, that the information-sharing capabilities of telemedicine enable access to remote experts to support medical decision-making on the scene.

In the past, the hospital staff knows about the patients transported by ambulances only when the patients arrived in the emergency department. In contrast, the 5G smart first-aid care platform informs the hospital of arriving patients in advance through functions such as one-click early warning for high-risk patients in front of the hospital, the green channel in the hospital, and emergency reception reminders. Especially for prehospital patients with severe trauma, when multidisciplinary treatment is needed, the functions of the 5G smart first-aid care platform enable hospital specialists to proceed to the emergency department ahead of time to perform various treatment preparations in advance, ensuring adequate time for the treatment of patients.

The 5G smart first-aid care platform supports docking with the in-hospital emergency system to provide first-aid patient electronic medical record data for the in-hospital emergency system. Emergency department medical staff can view the electronic medical record data of 120 prehospital patients in the emergency system. This platform realizes the electronic transfer in front of the hospital, assists medical staff to accurately diagnose patients, realizes homogenization management of prehospital medical documents, and finally realizes the whole process of quality control management of prehospital first-aid care.

For newly recruited emergency department medical staff, the 5G intelligence first-aid care platform can be used to cultivate hierarchical and multidimensional first-aid care skills by, for example, first, attentive observation of the hospital emergency command center; second, through on-the-spot learning, the development of a certain training cycle, and assessment standards; and finally, the realization of the homogenization of the emergency-care ability of medical staff.

## Prospect

**Figure 6:**
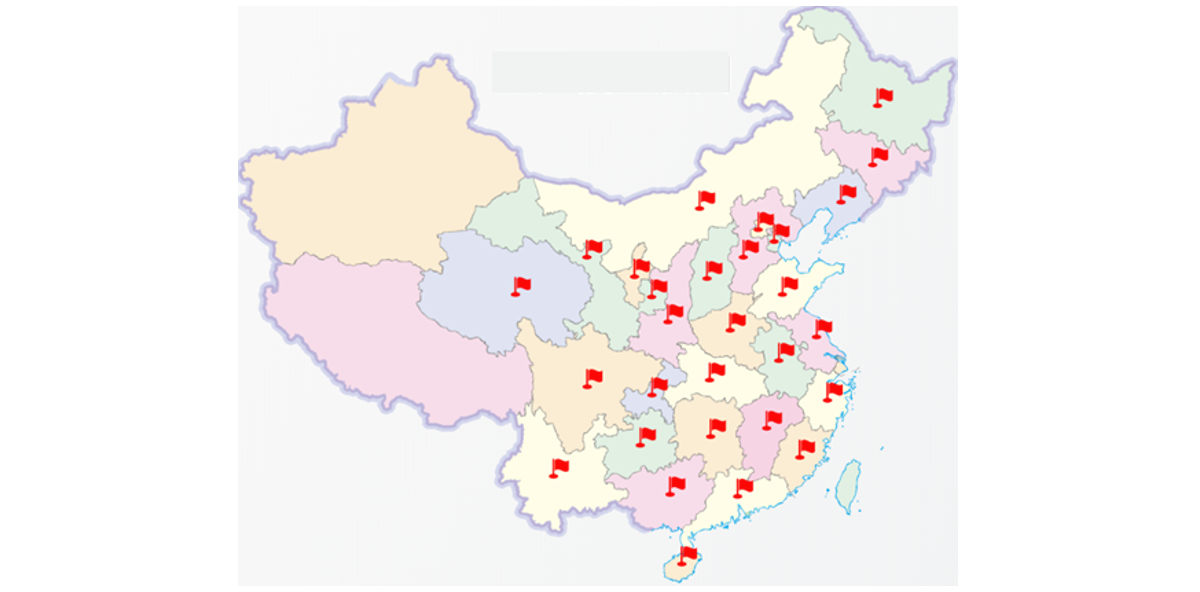
Distribution map of the current use of the 5G smart first-aid care platform in China. 5G: fifth generation.

Prehospital first-aid care combined with 5G technology is suitable for any prehospital first-aid mode, both domestically and abroad. At present, hospitals using the 5G smart first-aid care platform are spread across the country (Figure 6). This platform has completed >460,000 emergency-care tasks, uploaded >150,000 electrocardiograms, and accomplished 240,000 electronically signed informed consent forms. However, the application of the 5G smart emergency-care platform is at the stage of pilot exploration in large- and medium-sized cities. The cost of construction and technical support in the early stage is relatively expensive, so it is challenging to popularize and apply it in some areas with poor economic conditions. In addition, big data statistical analysis of the completed first-aid care tasks has not been performed yet. Therefore, the next work will focus on applying retrospective analysis methods to compare the patient outcome analysis, cardiopulmonary resuscitation success rate, and emergency medical quality analysis before and after the use of the 5G smart first-aid care platform to further verify its efficiency and safety.

## Conclusions

The 5G smart first-aid care platform realizes real-time interconnection of information between the ambulance and the hospital, performs remote consultation, moves the treatment “node” forward, shortens the treatment time, and enhances treatment efficiency. Future work should focus on larger-scale application and quality control analysis of the 5G smart first-aid care platform to fully explore its application.

## Abbreviations

5G: fifth generation
VR: virtual reality
